# Neurosurgery in a patient with severe hemophilia B: an experience using eftrenonacog alfa as perioperative management

**DOI:** 10.1002/ccr3.5848

**Published:** 2022-05-23

**Authors:** Olga Benitez Hidalgo, M. Fernanda Martinez Garcia, Agustin Bescos Cabestre, Juan Carlos Juarez Gimenez, Mercedes Gironella Mesa, Francesc Bosch Albareda

**Affiliations:** ^1^ Hematology Department Experimental Hematology Vall d'Hebron Institute of Oncology (VHIO) Hospital Universitari Vall d'Hebron Vall d'Hebron Barcelona Hospital Campus Barcelona Spain; ^2^ Medicine Department Universitat Autònoma de Barcelona Barcelona Spain; ^3^ Neurosurgery Department‐ Spine Unit Hospital Universitari Vall d’Hebron Barcelona Spain; ^4^ Hospital Pharmacy Department Hospital Universitari Vall d'Hebron Barcelona Spain

**Keywords:** anesthesia, hematology, neuosurgery, pharmacology, haemophilia B, rFIX‐Fc

## Abstract

Extended half‐life FIX (EHL‐FIX) concentrates have been developed with the purpose of reducing the frequency of infusions in patients with severe or moderate hemophilia B. We describe the case of a 63‐year‐old patient with severe hemophilia B (sHB) treated with FIX‐Fc fusion protein (rFIXFc) who underwent neurosurgery.

## Abstract 2

Hemophilia patients may need surgical interventions for complications either related or not to their coagulopathy. Real‐life experience is still limited using eftrenonacog alfa. This case demonstrates that it is an effective and safe treatment option.

## 1 INTRODUCTION

Hemophilia patients may need surgical interventions or invasive procedures for complications either related or not to their coagulopathy. These procedures require the intensified administration of concentrates of the deficient factor (FVIII in the case of hemophilia A and FIX in the case of hemophilia B) to reduce the bleeding risk associated with those procedures.^1^, ^2^

In recent years, new strategies have been developed for the prophylactic treatment of patients with hemophilia, such as extended half‐life factor concentrates (EHL). These products have shown improved pharmacokinetic properties, achieving parameters of half‐life (t_1/2_) 3‐ to 5‐fold longer in FIX EHL compared with standard FIX concentrates.^3^ This enables the extension of the dosing interval and allows for higher trough levels for longer periods of time.^2^

Studies with the different EHLs‐rFIX have shown that these products are effective and well‐tolerated in the perioperative management of patients with hemophilia.^4^ Nevertheless, real‐life experience as part of perioperative hemostatic management is still limited.

## 2 CASE REPORT

We report the case of a 63‐year‐old patient with severe hemophilia B (FIX <1 U/dl) on prophylactic treatment with standard half‐life recombinant FIX (SHL‐rFIX) twice a week (37 UI/kg/dose). This dosage was guided by a prior individual population pharmacokinetic (PopPK) study using the Web Accessible Population Pharmacokinetic Service‐Hemophilia (WAPPS‐Hemo) with a half‐life (t_1/2_) of 45.5 h, a clearance (Cl) of 0.31 L/h, and a volume of distribution at steady state (Vss) of 17.6 L. Concomitantly, the patient presents a bilateral rhizarthrosis and poor venous access, which makes self‐ treatment difficult. For this reason, he was switched to an extended half‐life FIX (EHL‐FIX), eftrenonacog alfa (rFIXFc). Treatment switch was also PK‐guided using (WAPPS‐Hemo).

The patient had been reporting low‐back pain, sciatica and gait disturbance for more than a year. The magnetic resonance imaging (MRI) reported severe stenosis of the lumbar canal at L4‐L5 level, with a mild L4‐L5 spondylolisthesis. The electromyography showed chronic L4‐L5‐S1 polyradiculopathy. Given the underlying coagulopathy and the risk of bleeding, surgical treatment through open laminectomy was recommended to maximize decompression and avoid future surgeries.

### 2.1 Hemostatic approach

Based on the information obtained in the individual population pharmacokinetic PopPK study, the patient started long‐term prophylaxis with rFIXFc every 14 days (93 IU/kg/14 days). Under this treatment regimen, the PK study showed a t_1/2_ of 128.5 h, a 0.143 L/h Cl, and a 22.6 L Vss.

Based on the baseline PK study, a dose of 93 IU/kg of rFIXFc was administered in bolus, prior to the intervention. Twelve hours after surgery, another 45 IU/kg bolus was subsequently administered. Complete blood counts (CBC) and FIX levels were assessed during the postoperative period as part of patient monitoring. The objective of the hemostatic treatment was to maintain FIX plasma levels over 80 IU/dl during the first 48 h post‐surgery. From Day +2 post‐surgery, a 45 IU/kg bolus was administered every 24 h to maintain levels between 60 and 80 IU/dl until the patient was discharged.

### 2.2 Surgical approach

A bilateral L4‐L5 approach was chosen, with a minimal opening spinal retractor through which an L4‐L5 laminectomy was done with partial medial facetectomy, achieving a wide decompression of the dural sac at L4‐L5 level. Bleeding during surgery was described as moderate by the surgeon (as expected for this type of surgery), achieving good bleeding control with bipolar coagulation and thrombin‐based hemostatic products, as is usually the case in non‐hemophilia patients during this type of procedure. A drain was left under the muscle layer.

No complications, including hemorrhage or infection, were reported during the postoperative period in the surgical area. The drain was removed three days after surgery, and the patient was discharged on the fourth day.

After discharge, the patient continued with hemostatic support with 45 IU/kg rFIXFc every 48h for four days, to maintain FIX levels above 50 IU/dL. Subsequently, at Day +14 post‐surgery, the patient had the stitches removed and received one administration at his usual dosage and continued with his standard regimen every 14 days.

## 3 DISCUSSION

### 3.1 Hemostatic approach discussion

Clinical trials with rFIXFc have shown that its half‐life is longer and its volume of distribution greater as compared with that of recombinant FIX SHL, while presenting an acceptable safety and efficacy profile in patients with severe hemophilia B.^5^, ^6^ Patients who received rFIXFc as prophylaxis, increased their dosage interval, mainly to a regimen of every 7 or 14 days.^5^, ^6^

According to the published data, after switching to rFIXFc, guided by PopPK, the patient decreased the frequency of administration, always maintaining trough levels above 4–5 IU/dl with his rFIXFc prophylaxis regimen every 14 days. Before the switch, the patient was on a twice weekly prophylaxis with rFIX, with trough levels between 2.5 and 3 IU/dl. The reduction in the frequency of administration is in line with an improvement in the PopPK profile of rFIXFc compared with the previous rFIX‐SHL, as described in clinical trials. According to the WAPPS‐Hemo estimation, the rFIXFc half‐life was 128.5 h compared with the previous half‐life of 45.5 h for rFIX‐SHL. This represents a 3‐fold increase in the half‐life of rFIXFc versus rFIX‐SHL.

### 3.2 Surgical approach discussion

Data have been published regarding the efficacy of rFIXFc in perioperative management, in both major and minor surgeries, in patients with hemophilia B within clinical trials. These studies suggest that rFIXFc is effective in perioperative management, providing FIX levels that are hemostatic enough in these cases, with lower consumption and less frequent dosing than with FIX SHL concentrates.^6^

Most patients received a median of 80–90 IU/kg bolus before the surgical procedure. Some patients required up to 2 doses of rFIXFc on the day of the surgery.^2^, ^6^, ^7^

Before surgery, our patient received a single bolus of 93 IU/kg of rFIXFc. This dose was similar to that from the surgeries described in clinical trials.^4^

In the postoperative period, our patient received 45 IU/kg rFIXFc bolus, every 12 h for the first 48 h after surgery, and 45 IU/kg every 24 h from Day +2 to Day +4 on which he was discharged. Total consumption including the pre‐surgery dose and the dose before the removal of stitches was 546 IU/kg. In the systematic literature review, we found no other cases with the same characteristics; therefore, it is not possible to compare data about consumption.^4^

In order to compare the use/effectiveness of rFIXFc *versus* rFIX‐SHL for the perioperative hemostatic management, we first estimated the doses of this product that would be necessary to maintain the desired trough levels, and then compared it with the doses used with rFIXFc. According to the WAPPS‐Hemo estimation, it would be necessary to administer a bolus of 100 IU/kg before surgery and subsequently prescribe 40 IU/kg every 8 h per day in the first 48 hours after surgery, and thereafter, the dosing interval could be lengthened to 40 IU/kg every 12 h. This data suggest that more frequent doses would be required with rFIX‐SHL in order to maintain the desired trough levels, which may lead to a longer hospital stay.

Due to our patient's history of hemophilia, lumbar decompression surgery without fusion was considered to minimize risk of bleeding, following the evidence of no need for fusion in grade I spondylolisthesis with spinal canal stenosis.^8^ Open laminectomy *versus* MIS decompression (minimally invasive spine procedure) was considered, and despite requiring greater exposure in open laminectomy, this surgery was chosen to maximize decompression and to avoid future new surgeries with its own risk of bleeding in each procedure.

The risk of intraoperative bleeding in this type of procedure is considered moderate, although postoperative bleeding after lumbar decompression carries risk of compression of the dural sac and neurological deficits in the lower limbs.

In the B‐LONG clinical trial, the mean doses after surgery (days 1–14 postoperatively) ranged between 49.12 and 64.61 IU/kg.^6^ At this stage, most patients received EHL‐FIX dosing approximately every 2 days to maintain the desired level of FIX activity depending on the type of surgery performed.^2^ No patient received daily dosing of FIX during the postoperative period, which comprises Days 0–14 post‐surgery.^6^

A drain was left under the muscle layer, and then, it was removed on the third day due to a serosanguineous discharge of 150 ml in 24 h, which is common in this type of open procedure. Our patient was discharged on the fourth postoperative day, which meant only one more day of admission than usual in general population. Contrary to what is indicated in the general population, the patient did not receive any antithrombotic treatment and did not present any type of thrombotic complication during or after his stay at the hospital.

No more intraoperative or postoperative bleeding was observed than those observed in the general population during this type of procedure, concluding that a good control of hemostasis was achieved.

rFIXFc treatment was well–tolerated, and no adverse events were observed. No inhibitor development against FIX was detected in subsequent controls.

## 4 CONCLUSION

In our experience in this case, rFIXFc is an effective and safe treatment option, allowing for a reduction in the number of infusions required to maintain an effective hemostatic perioperative control with the same length of hospital stay.

## AUTHOR CONTRIBUTIONS

Benitez Hidalgo, O; drafted the case report and approved all corrections made to it, including the final version to be published. Martínez García, MF; obtained patient's inform consent and critically revised the case report and made substantial corrections to all the drafts. Bescos Cabestre, A; reviewed literature on techniques and surgical approach and critically revised the case report and made substantial corrections to all the drafts. Juarez Gimenez, JC; supported regarding PK parameters and critically revised the case report and made substantial corrections to all the drafts. Gironella Mesa, M; Bosch Albareda, F critically revised the case report and made substantial corrections to all the drafts.

## CONFLICT OF INTEREST

None.

## ETHICAL APPROVAL

We confirm that the written informed patient consent has been collected for publication of this case report.

## CONSENT

We confirm that the written informed patient consent has been collected for publication of this case report.
